# Identification of potential biomarkers and pathways associated with carotid atherosclerotic plaques in type 2 diabetes mellitus: A transcriptomics study

**DOI:** 10.3389/fendo.2022.981100

**Published:** 2022-09-16

**Authors:** Tian Yu, Baofeng Xu, Meihua Bao, Yuanyuan Gao, Qiujuan Zhang, Xuejiao Zhang, Rui Liu

**Affiliations:** ^1^ Department of Very Important People (VIP) Unit, China-Japan Union Hospital of Jilin University, Changchun, China; ^2^ Department of Endocrinology, China-Japan Union Hospital of Jilin University, Changchun, China; ^3^ Department of Stroke Center, First Hospital of Jilin University, Changchun, China; ^4^ School of Stomatology, Changsha Medical University, Changsha, China

**Keywords:** type 2 diabetes mellitus, carotid atherosclerosis, transcriptome, biomarker, pathways, stable plaque, unstable plaque

## Abstract

Type 2 diabetes mellitus (T2DM) affects the formation of carotid atherosclerotic plaques (CAPs) and patients are prone to plaque instability. It is crucial to clarify transcriptomics profiles and identify biomarkers related to the progression of T2DM complicated by CAPs. Ten human CAP samples were obtained, and whole transcriptome sequencing (RNA-seq) was performed. Samples were divided into two groups: diabetes mellitus (DM) versus non-DM groups and unstable versus stable groups. The Limma package in R was used to identify lncRNAs, circRNAs, and mRNAs. Gene Ontology (GO) annotation and Kyoto Encyclopedia of Genes and Genomes (KEGG) pathway analyses, protein-protein interaction (PPI) network creation, and module generation were performed for differentially expressed mRNAs. Cytoscape was used to create a transcription factor (TF)-mRNA regulatory network, lncRNA/circRNA-mRNA co-expression network, and a competitive endogenous RNA (ceRNA) network. The GSE118481 dataset and RT-qPCR were used to verify potential mRNAs.The regulatory network was constructed based on the verified core genes and the relationships were extracted from the above network. In total, 180 differentially expressed lncRNAs, 343 circRNAs, and 1092 mRNAs were identified in the DM versus non-DM group; 240 differentially expressed lncRNAs, 390 circRNAs, and 677 mRNAs were identified in the unstable versus stable group. Five circRNAs, 14 lncRNAs, and 171 mRNAs that were common among all four groups changed in the same direction. GO/KEGG functional enrichment analysis showed that 171 mRNAs were mainly related to biological processes, such as immune responses, inflammatory responses, and cell adhesion. Five circRNAs, 14 lncRNAs, 46 miRNAs, and 54 mRNAs in the ceRNA network formed a regulatory relationship. C22orf34—hsa-miR-6785-5p—RAB37, hsacirc_013887—hsa-miR-6785-5p/hsa-miR-4763-5p/hsa-miR-30b-3p—RAB37, MIR4435-1HG—hsa-miR-30b-3p—RAB37, and GAS5—hsa-miR-30b-3p—RAB37 may be potential RNA regulatory pathways. Seven upregulated mRNAs were verified using the GSE118481 dataset and RT-qPCR. The regulatory network included seven mRNAs, five circRNAs, six lncRNAs, and 14 TFs. We propose five circRNAs (hsacirc_028744, hsacirc_037219, hsacirc_006308, hsacirc_013887, and hsacirc_045622), six lncRNAs (EPB41L4A-AS1, LINC00969, GAS5, MIR4435-1HG, MIR503HG, and SNHG16), and seven mRNAs (RAB37, CCR7, CD3D, TRAT1, VWF, ICAM2, and TMEM244) as potential biomarkers related to the progression of T2DM complicated with CAP. The constructed ceRNA network has important implications for potential RNA regulatory pathways.

## Introduction

Type 2 diabetes mellitus (T2DM) is a group of metabolic diseases that are mainly caused by chronic hyperglycemia due to multiple causes. Long-term progression can lead to atherosclerotic cardiovascular disease (ASCVD), which is the main cause of death in T2DM patients (1). When atherosclerosis (AS) occurs, a series of pathological changes, including fibro-lipopathy and foam cell necrosis, occur on the arterial wall and lead to plaque formation (2, 3). Cervical plaque formation is one of the main causes of stroke (4). The American Heart Association classifies carotid atherosclerotic plaques (CAPs) histologically into stable and unstable plaques (5). The formation and progression of unstable CAPs lead to more dangerous ischemic stroke events (6).

Patients with T2DM are more likely to form unstable plaques, and the probability of stroke is twice that of those without T2DM (7). Thus, increased vulnerability to CAP development in T2DM patients may be due to aggravated inflammation (8, 9), increased neovascularization (8), promotion of liponuclear expansion (10, 11), growing numbers of plaques (12), and other mechanisms. Clinically, it is of great significance to identify the stability of CAPs in T2DM patients as early as possible and to actively prevent the formation of unstable plaques. Unfortunately, plaque stability cannot be determined histologically despite the increasing number of methods to detect the development of CAPs in T2DM patients. Earlier bioinformatics studies have reported that T2DM alters CAP gene expression (13), but this has not been further confirmed with transcriptomics. Consequently, key biomarkers for T2DM complicated by CAP progression are currently unavailable.

In our study, after a carotid endarterectomy, CAPs were sampled for histological classification and high-throughput sequencing. Using bioinformatics analysis, we determined the corresponding biomarkers at the transcriptome level that indicate the stability of CAPs in T2DM patients. Additionally, the pathogenesis of the disease was explored and treatment targets are discussed.

## Materials and methods

### Data source

Samples were collected from 10 patients with CAPs at the First Hospital of Jilin University (Changchun, Jilin, from July 2019 to November 2019). The procedure was approved by the Ethics Committee of the First Hospital of Jilin University (No. 2019-272), and written informed consent was obtained from each participant. The processes of tissue classification, storage, transportation, RNA extraction, library preparation, RNA sequencing, and identification of mRNA, lncRNA, and circRNA spectra are consistent with our previously published research (14). Similarly, the transcriptome data PRJNA752896 from this study can be found in the NCBI database (https://submit.ncbi.nlm.nih.gov/subs/bioproject/).

We used “atherosclerotic plaque” and “type 2 diabetes” as keywords to search for relevant information in GEO (http://www.ncbi.nlm.nih.gov/geo) (15). The GSE118481 dataset satisfied our research conditions. This dataset included 16 samples of non-T2DM with CAPs (six asymptomatic and 10 symptomatic) and eight samples of T2DM with CAPs (six asymptomatic and two symptomatic). See **Supplementary Figure 1** for a flowchart.

### Distribution and comparative analysis of expression abundance among samples

A variety of methods were used to evaluate the correlations among 10 samples, using the cor function in R version 3.6.1 (https://stat.ethz.ch/R-manual/R-devel/library/stats/html/cor.html) to calculate the Pearson’s correlation coefficient (PCC) between every two samples. The closer the correlation coefficient is to 1, the higher the similarity of expression patterns between samples (16), using version 1.7.8 of the psych package (https://cran.r-project.org/web/packages/psych/index.html) in R to perform principal component analysis (PCA) based on expression abundance on all samples to view the distribution among samples.

### Screening of significantly differentially expressed RNAs and enrichment analysis of mRNAs

The Limma package (version 3.32.5) (17) (http://bioconductor.org/packages/release/bioc/html/limma.html) in R was used to screen RNAs (including lncRNA, circRNA, and mRNA) with a significant difference between the two comparison groups. FDR less than 0.05 and |log2FC|>0.5 were selected as the threshold criteria for screening significant differences. For the significantly differentially expressed RNAs screened from the two groups, the heatmap package (version1.0.8) (18) (https://cran.rproject.org/web/packages/pheatmap/index.html) in R was used to create a two-way hierarchical clustering heatmap.

Subsequently, the sets of significantly differentially expressed lncRNAs, circRNAs, and mRNAs screened from the two comparison groups of DM versus non-DM and unstable versus stable were compared, and the common RNAs of the two comparison groups were obtained. Next, the directions of the significant differences were investigated, and those that differed in the same direction among the common RNAs were retained as the target objects of future research. The mRNAs in the retained RNAs were enriched and analyzed based on Gene Ontology (GO) and Kyoto Encyclopedia of Genes and Genomes (KEGG) (19) using the DAVID (version 6.8) (20, 21) online analysis tool (https://david.ncifcrf.gov/), and a p-value less than 0.05 was selected as the screening significance threshold for correlations. The GO terms include biological process (BP), cellular component (CC), and molecular function (MF) (22).

### Construction of protein-protein interaction (PPI) network

The STRING database (version11.0) (23) (http://string-db.org/) was used to search the interactive relationships between the proteins at the intersection of significantly differently expressed gene products retained in the two comparison groups. The interaction network was constructed, and then a visual representation was generated using Cytoscape (version 3.9.0) (24)(http://www.cytoscape.org/). The centrality of the gene nodes in the network was calculated using the plug-in CentiScaPe (version 2.2) (25) (http://apps.cytoscape.org/apps/centiscape) of Cytoscape. The three most common methods for measuring node centrality are degree centrality (DC), closeness centrality (CC), and betweenness centrality (BC). Next, the important gene nodes were screened by the centrality parameter, and the closely connected genes in the network were obtained. Then, the module identification plug-in MCODE (version1.4.2) in Cytoscape was used to partition and identify the network modules (parameters: degree cutoff=2, node score cutoff=0.2, k-core=2). Another plug-in, BINGO (version 2.44; http://apps.cytoscape.org/apps/bingo; FDR<0.05) (26), was used to divide and annotate the functional modules.

### Construction of transcription factors(TFs)-differentially expressed gene regulatory network

The Translational Regulatory Relationships Unraveled by Sentence-based Text Mining (TRRUST) database (27) (https://www.grnpedia.org/trrust/) establishes the transcriptional regulation relationship of TFs based on literature mining. We uploaded the genes with significant differential expression at the intersection screened by the two comparison groups to the database and selected TFs with regulatory connection relationships with the genes with significant differential expression at the intersection. Next, according to the relationship between TFs and regulatory genes, a regulatory network was constructed and visually displayed using Cytoscape (24).

### Construction of co-expression network of circRNA-mRNA and lncRNA-mRNA

For the significantly differentially expressed lncRNAs, circRNAs, and mRNAs screened from the two comparison groups, the PCC (28) between the expression levels of circRNA-mRNA and lncRNA-mRNA was calculated using the cor.test function in R. Connection pairs with a p-value less than 0.05 were screened, and co-expression networks of circRNA-mRNA and lncRNA-mRNA were constructed. The networks were visualized using Cytoscape (24).

### Construction of competitive endogenous RNA(ceRNA) network

To construct lncRNA/circRNA-miRNA relationships, the sequences of all human miRNAs were downloaded from miRBase (https://www.mirbase.org/), and then the sequences of significantly differentially expressed lncRNAs and circRNAs were extracted and retained by the two comparison groups from the RNA-seq data. Subsequently, the miRanda localization tool (29) (http://cbio.mskcc.org/miRNA2003/miranda.html) was used to predict the binding relationships of lncRNA-miRNA and circRNAs-miRNA (miRanda alignment parameter settings: gap extend=0, score threshold =100, energy threshold =-20, % matched seq threshold =80%).

For the construction of miRNA-mRNA relationships, the miRNAs linked to lncRNAs and circRNAs in the previous step were searched and the target genes they regulated were identified using the miRwalk3.0 database (30)(http://mirwalk.umm.uni-heidelberg.de/). Further, the significantly differentially expressed mRNAs screened from the two comparison groups were mapped to the target genes to identify the significantly differentially expressed mRNAs that were regulated.

To construct a ceRNA network, the lncRNA/circRNA-miRNA-mRNA regulatory network was formed after integration based on the relationships between lncRNA/circRNA-miRNA and miRNA-mRNA. The generated network was visualized using Cytoscape (24).

### Public database validation analysis

The gene expression profile data in the GSE118481 dateset were downloaded from the GEO database, which included 24 CAP tissue samples. Further, all samples were divided into two comparison groups according to source information: DM versus non-DM and stable versus unstable. The same Limma algorithm and screening threshold (FDR is less than 0.05, |log2FC|>0.5) were used, and mRNAs with significant differential expression in the two comparison groups were screened. Next, we compared the mRNAs screened from the two comparison groups in the RNA-seq data, selected the overlapping sections, and visualized the mRNA expression levels of the overlapping sections in the RNA-seq data and GEO data.

### Reverse-Transcription quantitative polymerase chain reaction (RT-qPCR) validation

We used seven unstable CAPs with DM and seven stable CAPs without DM for liquid nitrogen grinding and Trizol reagent (Invitrogen, Carlsbad, California, USA) extraction to obtain total RNA. After concentration and quality evaluation by microplate reader, total RNA was reverse transcribed to cDNA using the RevertAid First Strand cDNA Synthesis Kit (Thermo Scientific, Waltham, Massachusetts, USA). Then, the cDNA samples were mixed with FastStart Universal SYBR Green Master (Rox) (Roche, Mannheim, Germany) and injected into an Eppendorf AG 22331 Hamburg PCR Thermal Cycler. β-Actin was used as the internal control. Primer sequences are listed in **Supplementary Table 1**. All samples were run in triplicate, and the results were analyzed using the 2(−ΔΔCt) method.

### Construction of regulatory network where important factors are located

The overlapping mRNAs obtained in the previous step, were combined with the PPI network, TF-mRNA regulatory network, lncRNA/circRNA-mRNA co-expression network, and the ceRNA regulatory network previously constructed. Then, the overlapping mRNAs were extracted to jointly build a comprehensive network.

### Statistical analyses

GraphPad Prism was used to draw graphics and perform statistical analysis of RT-qPCR data using the Wilcoxon rank sum test. Other statistical analyses were performed using R software. T-tests were used to compare differences in expression between the groups. The expression level of mRNAs is presented as the mean ± SD. *p* < 0.05 was considered statistically significant.

## Results

### Distribution and comparative analysis of expression abundance

**Figure 1:**
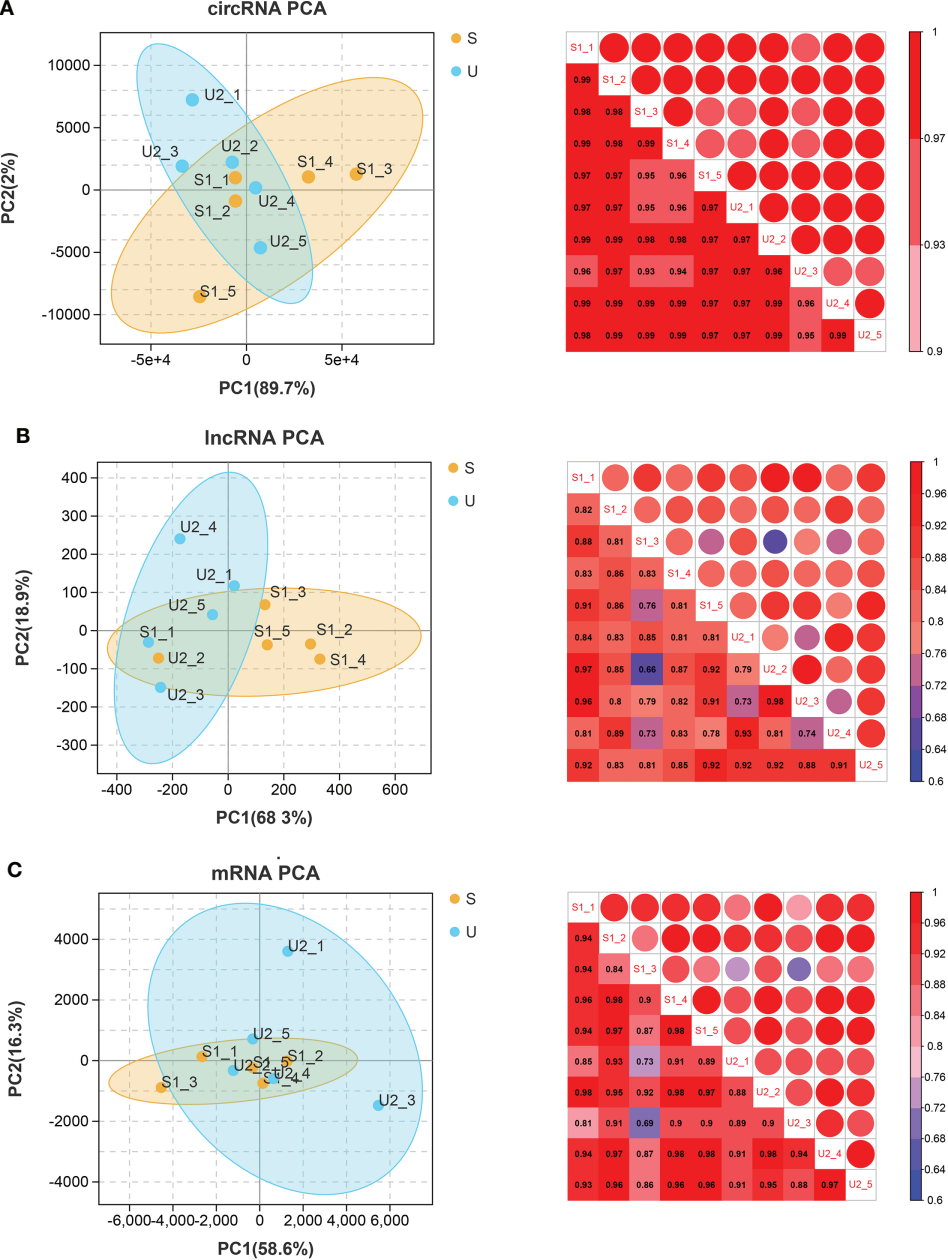
circRNA **(A)**, lncRNA **(B)**, and mRNA **(C)**. Left: PCA map of samples based on expression abundance. X, Y, and Z axes represent PC1, 2, and 3 respectively. Dots of different colors represent samples of different groups (S represents the stable group and U represents the unstable group); Right: heatmap of correlation between two samples based on expression abundance. The change of color from cold color to hot color indicates the relationship of the correlation coefficient between samples from small to large, the horizontal and vertical axes represent the name of samples, and the numbers in each grid represent the value of correlation coefficient.

The PCCs between samples with different RNAs expression levels were distributed above 0.6. PCA analysis was performed based on expression abundance. The results showed that the correlation between gene expression levels was very high, the sample distribution was relatively clustered, and there was no discrete type (**Figure 1**).

### Identification of differentially expressed RNAs

**Figure 2:**
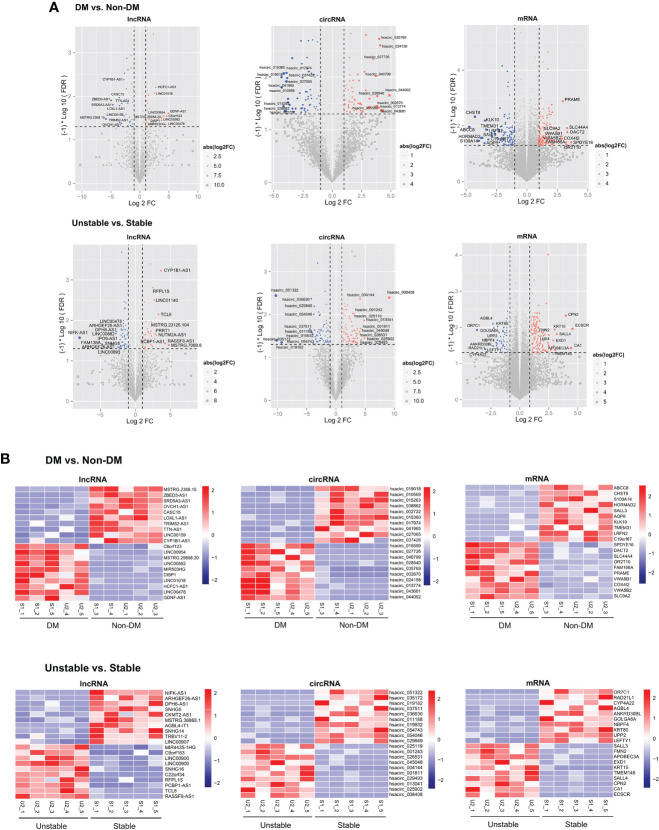
**(A)** The DM versus non-DM and unstable versus stable comparison groups volcano plot. The blue and red dots respectively indicated significantly downregulated and upregulated RNAs, the horizontal dotted line indicated FDR<0.05, and the two red vertical dotted lines indicated |log2FC|>0.5. The top 10 RNAs are marked. **(B)** Heat map of the expression levels of the top 10 RNAs in DM versus non-DM and unstable versus stable groups.

Statistics on the number and types of significantly differentially expressed RNAs were screened according to the two comparison groups and the common RNAs following comparison are shown in **Supplementary Table 2**. Among them, 180 lncRNAs, 343 circRNAs, and 677 mRNAs were differentially expressed between the DM versus non-DM groups, and 240 lncRNAs, 390 circRNAs, and 677 mRNAs were differentially expressed between the unstable versus stable groups. After investigating the direction of the significant differences of overlapping RNAs in the two comparison groups, five circRNAs (three downregulated and two upregulated), 14 lncRNAs (five downregulated and nine upregulated), and 171 mRNAs (80 downregulated and 91 upregulated) had the same significant difference directions in the two comparison groups. **Figure 2A** shows volcano plots depicting the significantly differentially expressed RNAs and whether they were up- or downregulated. Heatmaps of the expression level of each of the top 10 RNAs that were up- and downregulated among the significantly differentially expressed RNAs are shown in **Figure 2B**.

### Function and pathway enrichment analysis of differentially expressed mRNAs

**Figure 3:**
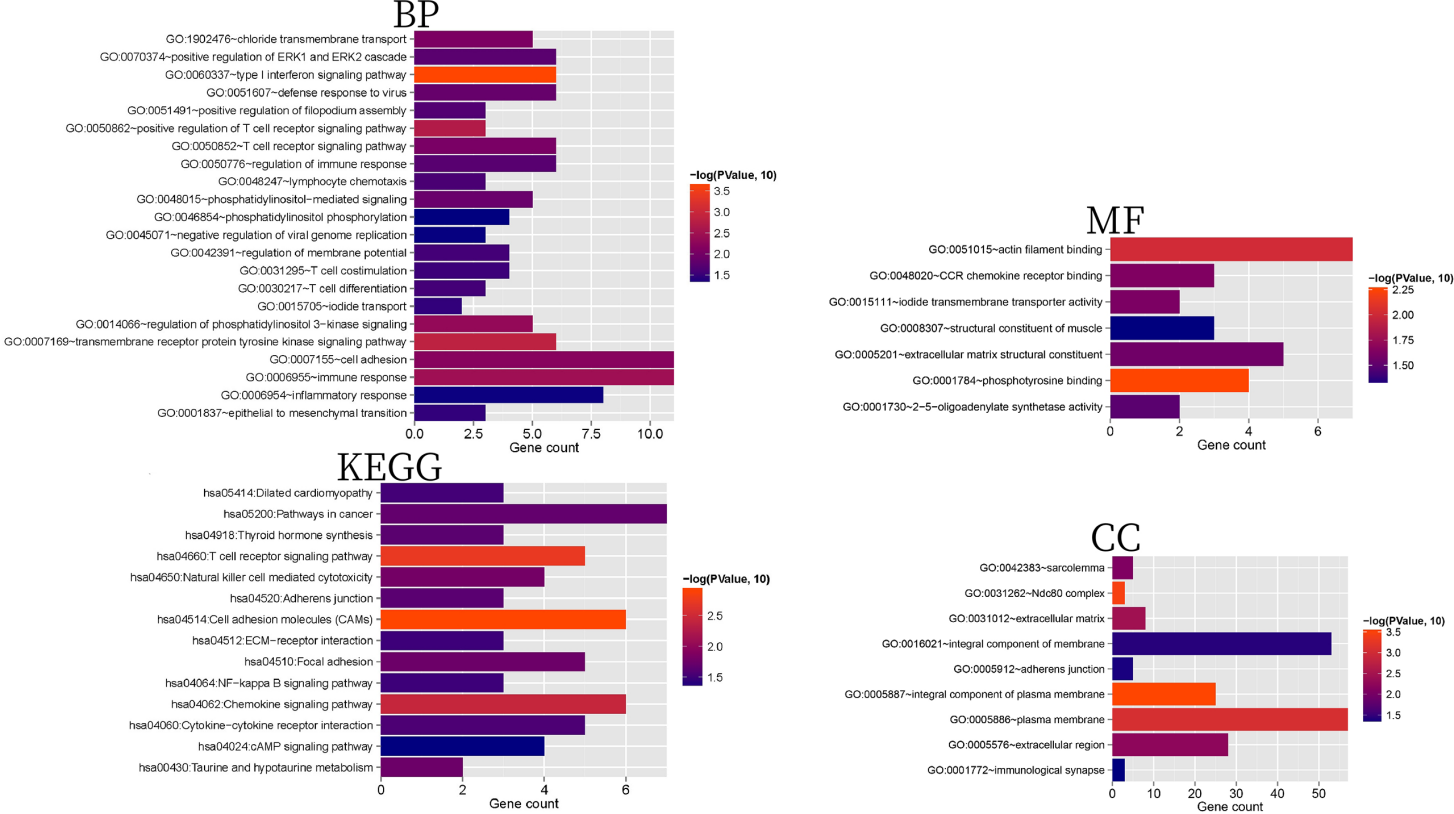
BP, CC, MF, and KEGG histograms related to 171 mRNAs with significant differential expression. The horizontal axis represents the number of genes, the vertical axis represents the significantly related items, and the color represents the correlation. The closer to red, the higher the significance.

We screened 22 BPs, nine CCs, seven MFs, and 14 KEGG signaling pathways (**Supplementary Table 3**); the columnar figure is shown in **Figure 3**. The results showed that BPs and KEGG pathways of 171 mRNAs were significantly enriched in positive regulation of T cell receptor signaling pathway, regulation of immune response, lymphocyte chemotaxis, T cell differentiation, T cell costimulation, inflammatory response, cell adhesion molecules (CAMs), chemokine signaling pathway, focal adhesion, adherens junction, and NF-kappa B signaling pathways, which are involved in immune responses, cell adhesion, and inflammatory reactions. Additionally, the type I interferon signaling pathway and epithelial to mesenchymal transition are considered to be related to the progression of AS (31, 32), and regulation of phosphatidylinositol 3-kinase signaling is considered to be related to insulin resistance and AS (33). MFs were significantly enriched in phosphotyrosine binding, actin filament binding, CCR chemokine receptor binding, iodide transmembrane transporter activity, extracellular matrix structural constituent, 2’-5’-oligoadenylate synthetase activity, and structural constituent of muscle. CCs were significantly enriched in the integral component of plasma membrane, Ndc80 complex, extracellular matrix, extracellular region, sarcolemma, integral component of membrane, adherens junction, and immunological synapse.

### Construction of a PPI network of differentially expressed mRNAs, regulatory relationship with TFs, co-expression network with differentially expressed circRNAs, and differentially expressed lncRNAs

**Figure 4:**
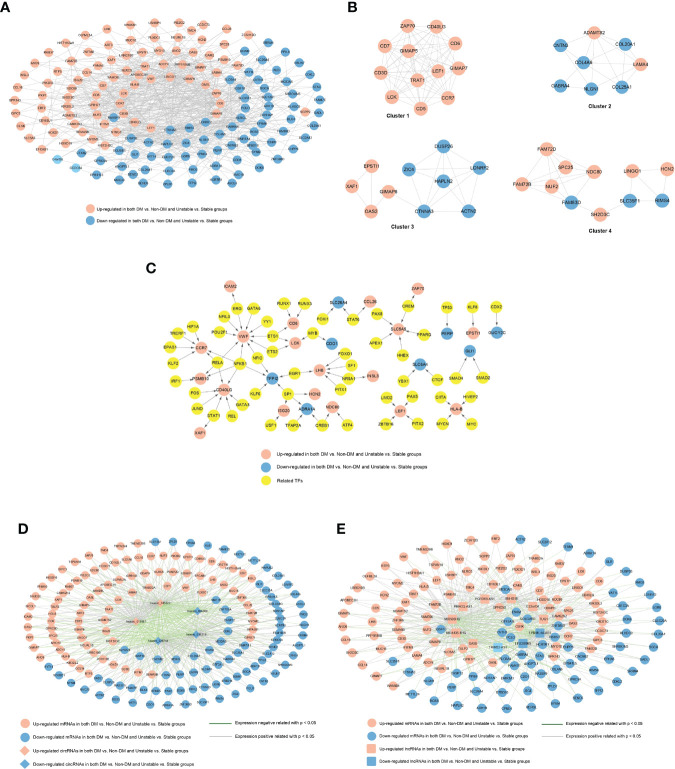
**(A)** PPI network of significantly differentially expressed genes. The red and blue nodes represent the genes that are significantly upregulated and downregulated, respectively, in the two comparison groups. **(B)** Interaction network module diagram. The red and blue nodes represent the genes that are significantly upregulated and downregulated, respectively, in the two comparison groups. **(C)** TF-differential gene regulatory network. The yellow square and circle represent TFs and significantly differentially expressed genes, respectively, and the red and blue nodes represent the genes that are significantly upregulated and downregulated, respectively, in the two comparison groups. **(D)** circRNAs-miRNA co-expression network, red and blue represent the RNAs that are significantly upregulated and downregulated, respectively, in the two comparison groups, and round and diamond represent mRNAs and circRNAs, respectively; The blue and gray lines indicate significant negative and positive correlations, respectively. **(E)** lncRNA-miRNA co-expression network, red and blue represent the RNAs that are significantly upregulated and downregulated, respectively, in the two comparison groups, and round and square respectively represent mRNAs and lncRNAs; The blue and gray lines indicate significant negative and positive correlations, respectively.

A total of 697 pairs of interaction links were obtained using the STRING database to construct an interaction network. As shown in **Figure 4A**, the network contained 154 gene nodes. The important connecting genes in the network were screened and sorted from high to low according to the degree of nodes; the top 20 are displayed in **Supplementary Table 4**. The top 20 genes were LONRF2, CTNNA3, VWF, LCK, CCR7, LEF1, CD3D, GIMAP8, OASL, RIMS4, GIMAP5, HLA-B, LINGO1, ZAP70, CD40LG, CD5, TRAT1, ACTN2, HAPLN2, and DUSP26.

The PPI network was divided into four modules (**Figure 4B**). After GO annotation, we obtained BP, MF, and CC which were significantly related to each module. Among the BPs, modules 1, 2, 3, and 4 were significantly related to lymphocyte activation, extracellular structure organization, RNA catabolic process, and nuclear division, respectively. Among the MFs, they were each involved in one of the armadillo repeat domain binding, neurotransmitter receptor activity, transcription cofactor activity, and protein domain-specific binding processes. Among the CCs, they were involved in the cell surface, extracellular matrix, and nucleus. All the significantly relevant GO annotations for each module are listed in **Supplementary Table 5**.

A total of 88 TF-mRNA regulatory junction pairs were screened. Based on the relationships between TFs and regulatory genes, a regulatory network was constructed (**Figure 4C**). We also compared all TFs in the TRRUST database with 171 target mRNAs and obtained four intersections (Dlx4, Gli1, LEF1, and hoxd1).

A total of 516 and 450 connecting pairs were screened from circRNA-mRNAs and lncRNA-mRNAs, respectively, to construct the co-expression network of circRNA-mRNAs and lncRNAs-mRNAs (**Figures 4D, E**).

### Construction of ceRNA network

**Figure 5:**
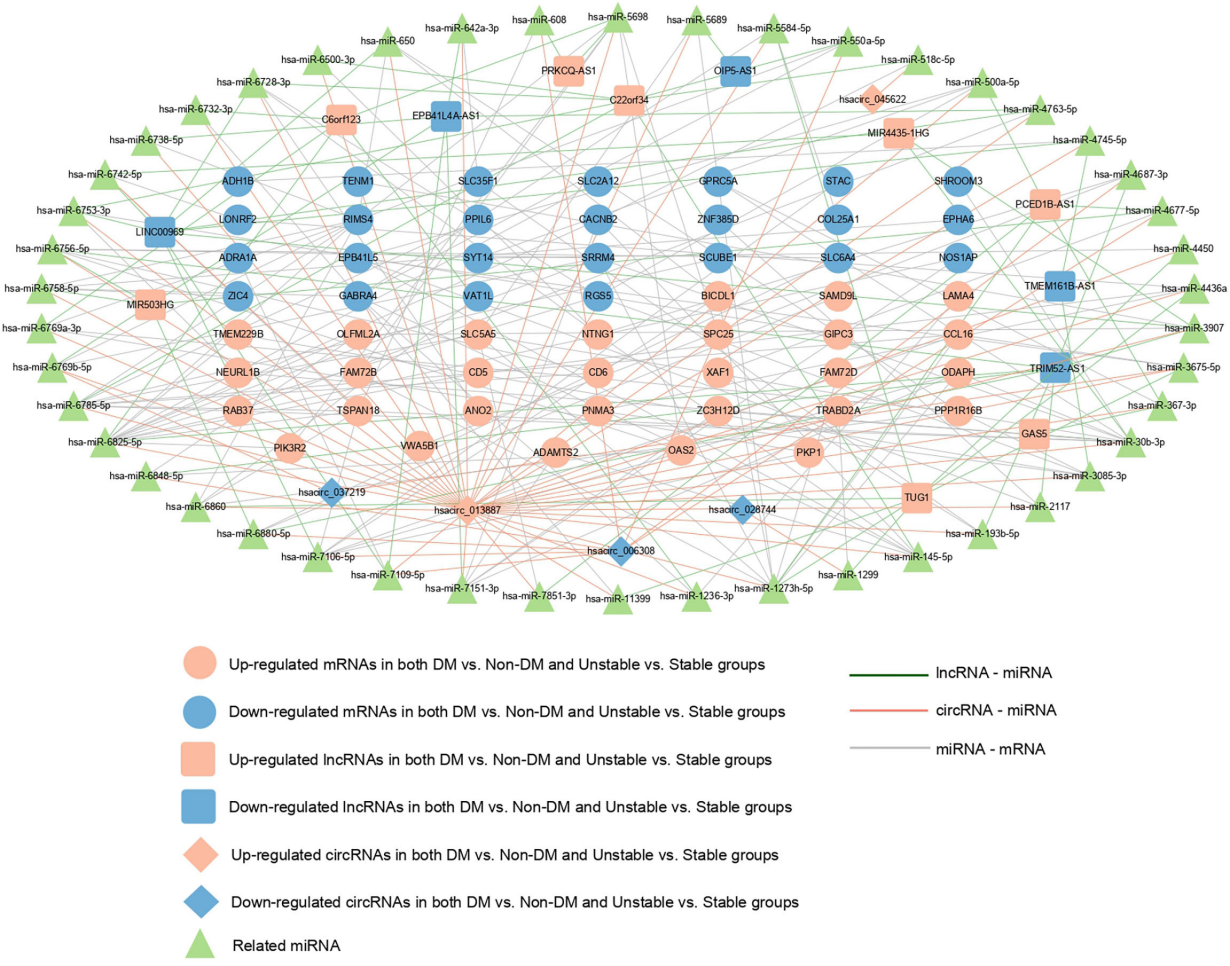
ceRNA regulatory network: red and blue represent the RNAs that are significantly upregulated and downregulated, respectively, in the two comparison groups. Round, diamond, and square respectively represent mRNAs, circRNAs, and lncRNAs, and green triangle represents the related miRNAs. Blue, red, and gray represent lncRNA- miRNA, circRNAs-miRNA, and miRNA-mRNA connections, respectively.

A total of 260 circRNAs-miRNA, 612 lncRNA-miRNA, and 131 miRNA-mRNA linkage pairs were obtained. Based on these regulatory relationships, a ceRNA regulatory network integrating the circRNA/lncRNA-miRNA regulatory axis was constructed (**Figure 5**). The network included five circRNAs, 13 lncRNAs, 46 miRNAs, and 54 mRNAs.

### GSE118481 dataset and RT-qPCR validation analysis

**Figure 6:**
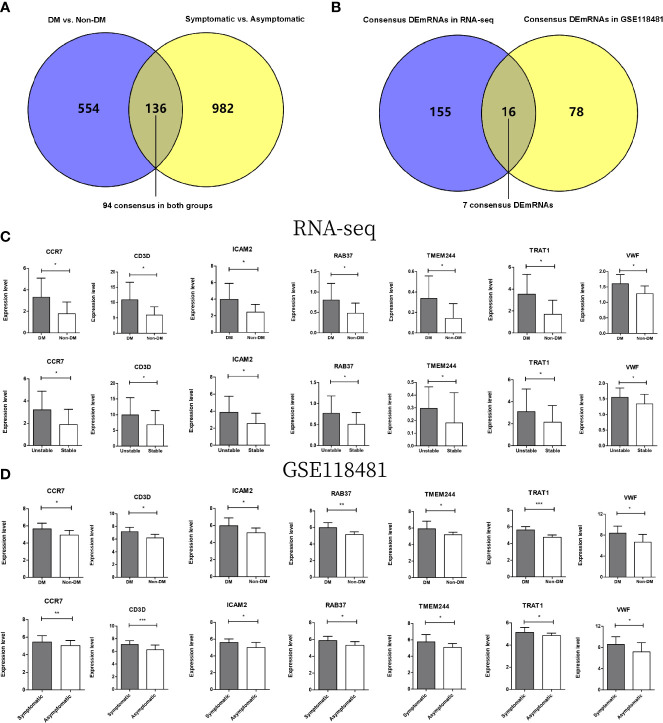
**(A)** Wayne diagram for comparison of significantly differentially expressed genes in DMs versus non-DM and symptomatic versus asymptomatic groups in GSE118481 datasets. **(B)** Wayne diagram comparing overlapping genes in DM versus non-DM and stable versus unstable groups with target mRNA in RNA-seq data results. **(C)** The expression levels of 7 genes were distributed in two comparison groups in the RNA-seq data. **(D)** The expression levels of 7 genes were distributed in two comparison groups in the GSE118481 dataset. * indicates *p*<0.05, * * indicates *p*<0.01, * * * indicates *p*<0.001.

A total of 690 and 1118 significantly differentially expressed genes meeting the threshold requirements were screened in the DM versus non-DM and symptomatic versus asymptomatic groups, respectively. A total of 136 overlapping regions were screened in both groups (**Figure 6A**). Then, the direction of significant differences in overlapping genes in the two comparison groups was investigated. A total of 94 significantly differentially expressed genes with the same direction of difference were obtained.

After comparing these 94 genes with 171 mRNAs screened from the two comparison groups of RNA-seq data (**Figure 6B**), a total of 16 overlapping genes were obtained, of which seven genes were in the same direction of difference in the RNA-seq data and GSE118481 data (CCR7, CD3D, ICAM2, TMEM244, TRAT1, VWF, and RAB37). Column charts of the expression levels of the seven genes in different data are shown in **Figures 6C, D**.

**Figure 7:**
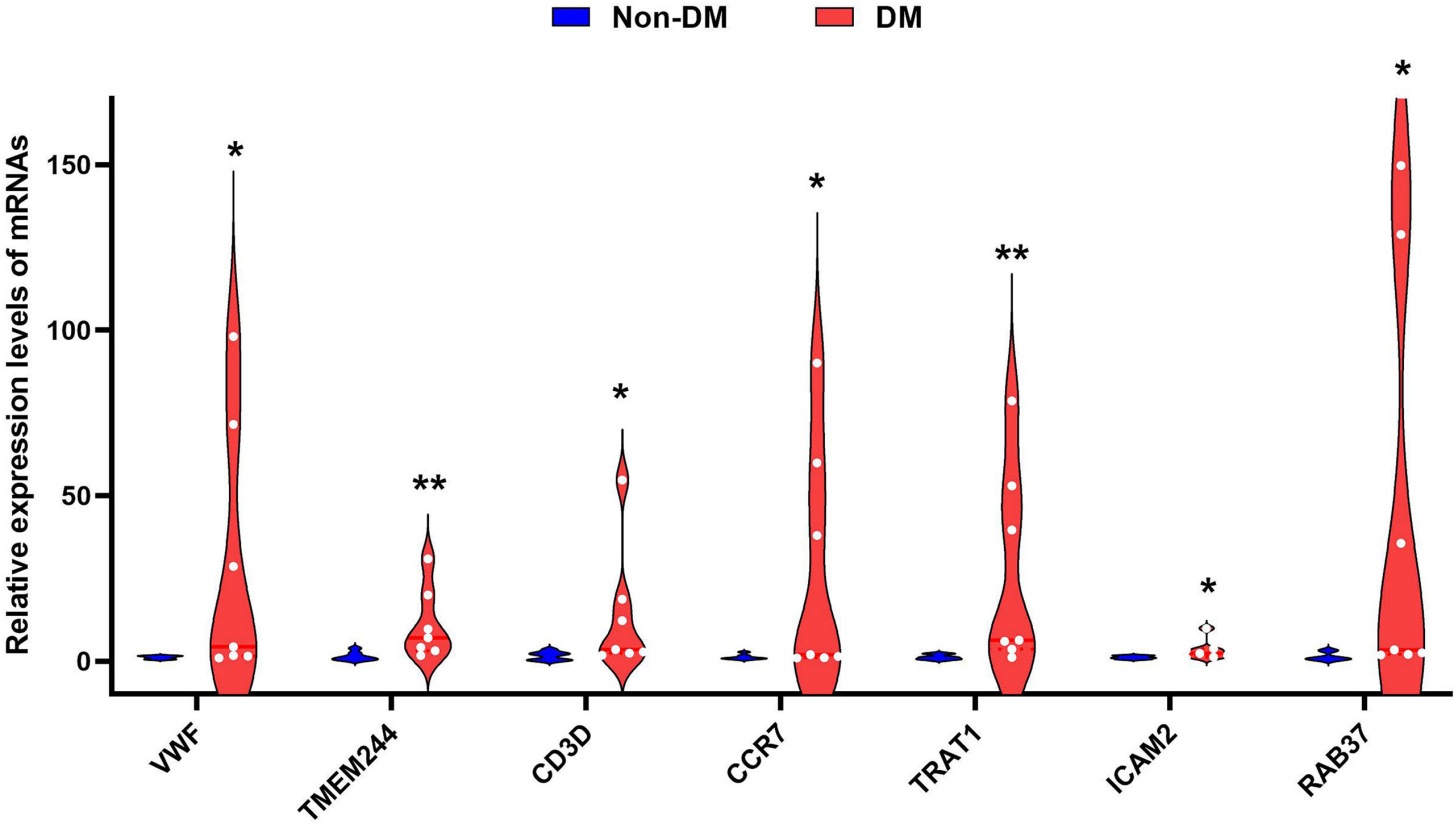
Violin diagram of RT-qPCR results for seven genes. * indicates p < 0.05, ** indicates p < 0.01.

RT-qPCR verified that CCR7 (p < 0.05), CD3D (p < 0.05), ICAM2 (p < 0.05), TMEM244 (p < 0.01), TRAT1 (p < 0.01), VWF (p < 0.05), and RAB37 (p < 0.05) were upregulated in the DM with unstable CAP group compared with the non-DM with stable CAP group (**Figure 7**).

### Construction of a regulatory network where the seven mRNAs are located

**Figure 8:**
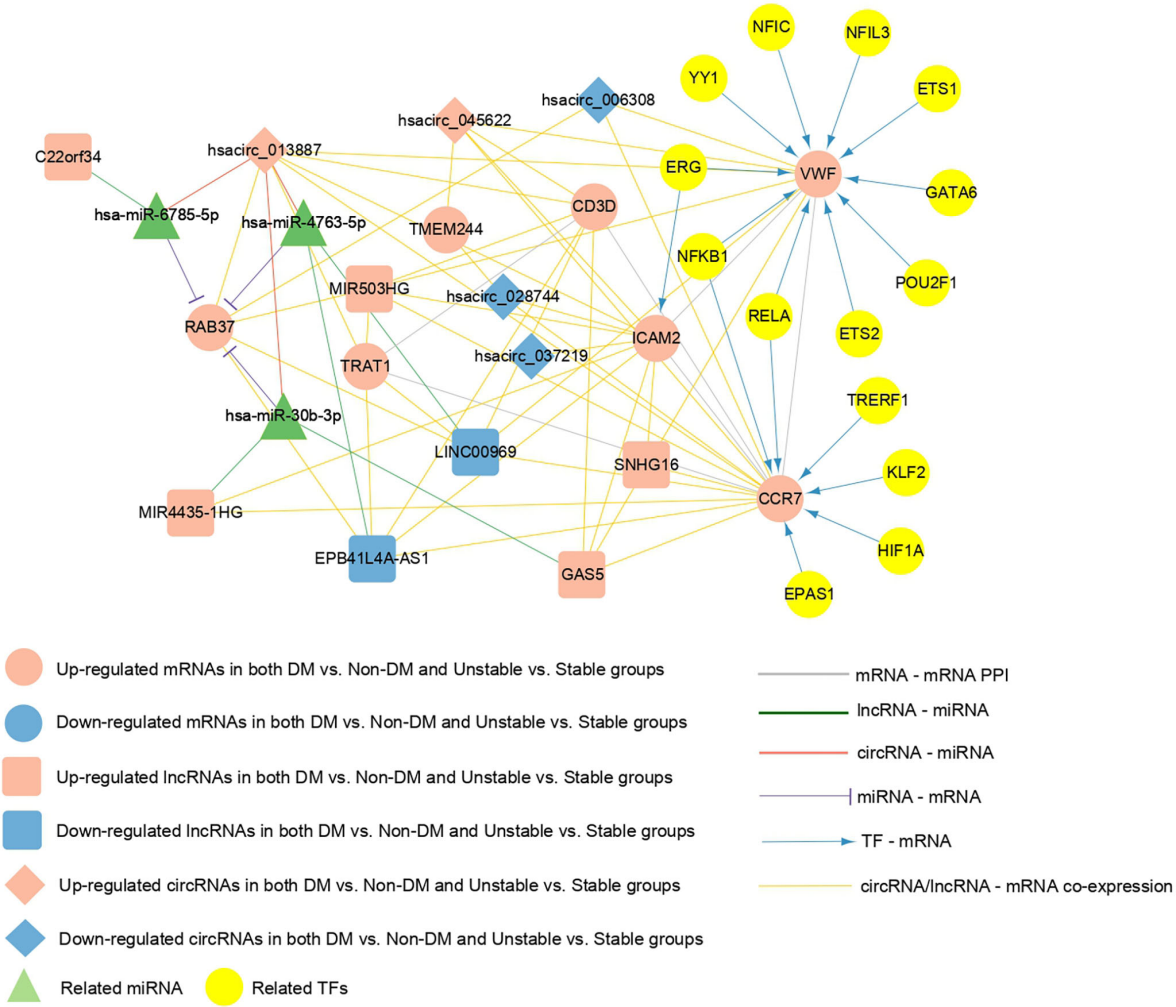
The comprehensive network of important overlapping genes. Red and blue represent the RNAs that are significantly upregulated and downregulated, respectively, in the two comparison groups. Circles, diamonds, and squares represent mRNAs, circRNAs, and lncRNAs, respectively, green triangles represent related miRNAs, and yellow circles represent TFs. Grey lines indicate an interaction connection. Green, red, and purple line respectively indicate lncRNA-miRNA, circRNA-miRNA, and miRNA- mRNA connections. Blue lines indicate TF-mRNA connections, and yellow lines indicate circRNA/lncRNA-mRNA co-expression associations.

Crucial factors related to seven mRNAs were extracted from the PPI network, TF-mRNA regulatory network, lncRNA/circRNAs-mRNA co-expression network, and the ceRNA regulatory network previously constructed. **Supplementary Table 6** shows the connection relationships used to build a comprehensive network where the overlapping important mRNAs are located (**Figure 8**). The important factors included in the network were as follows: five circRNAs (hsacirc_028744, hsacirc_037219, hsacirc_006308, hsacirc_013887, and hsacirc_045622), six lncRNAs (EPB41L4A-AS1, LINC00969, GAS5, MIR4435-1HG, MIR503HG, and SNHG16), and seven mRNAs (RAB37, CCR7, CD3D, TRAT1, VWF, ICAM2, and TMEM244). Among them, TFs including ERG, ETS1, ETS2, GATA6, NFIC, NFIL3, NFKB1, POU2F1, POU2F1, RELA, and YY1 are involved in the regulation of the gene VWF; TFs including EPAS1, HIF1A, KLF2, NFKB1, RELA, and TRERF1 are involved in the regulation of the gene CCR7; and the TF of ERG is involved in the regulation of the gene ICAM2. In addition, C22orf34 can act as a ceRNA to compete with RAB37 through hsa-miR-6785-5p, hsacirc_013887 can compete with RAB37 through hsa-miR-6785-5p, hsa-miR-4763-5p, hsa-miR-30b-3p, MIR4435-1HG can compete with RAB37 through hsa-miR-30b-3p, and GAS5 can compete with RAB37 through hsa-miR-30b-3p. These six circRNA/lncRNA-miRNA-mRNAs may be key regulatory axes in the ceRNA network.

## Discussion

Patients with T2DM are more likely to face CAP-related complications than those without T2DM (7). These T2DM patients are also more likely to develop unstable AS plaques, resulting in more serious cardiovascular events (34). Therefore, it is crucial to identify the T2DM complicated by unstable CAP earlier in clinical practice. Further, mechanisms underlying disease progression need to be elucidated along with identification of corresponding therapeutic targets.

By comparing the genes expressed in human T2DM patients with unstable CAP samples and non-T2DM patients with stable CAP samples, we identified a total of 171 target mRNAs with significant differential expression, of which 91 were upregulated and 80 were downregulated. By applying GO annotation and KEGG pathway analyses, we noticed that most of the enriched items were related to immune responses, cell adhesion, and inflammatory responses, which is consistent with published studies (3, 35–40). Further analyses indicated that AS formation cannot be separated from the adhesion and migration of monocytes, inflammatory cells, inflammatory mediators, and cytokines in the arterial wall (3). The enrichment results also showed that the plaque formation process of T2DM with unstable CAP also involves a similar pathogenesis. T2DM exacerbates the direct effect of inflammation on AS (35), and the immune response also plays an important role in the progression of AS (36, 37). Adaptive and innate immunity play an important role in the progression of T2DM (38, 39), and hyperglycemia promotes AS progression by regulating the adaptive immunity of macrophages (40). The microenvironment of AS plaques is very complex, where both inflammatory and immune responses are involved and play an important role (41–43). Considering that immune cell infiltration plays a key role in CAP development (44, 45) and that hyperglycemia alters the plaque environment, it is surprising that the role of immune cell infiltration in the progression of CAP under hyperglycemia remains unexplored. Future research should address this issue. Furthermore, research on the CAP microenvironment and the emergence of corresponding therapeutic targets provide a new research direction for the therapies of T2DM complicated with CAP (42, 43, 46, 47).

We identified seven potential mRNAs, five circRNAs, and six lncRNAs using comprehensive bioinformatic methods. These genes and non-coding RNAs may serve as biomarkers for CAP progression in T2DM. Among them, CCR7, ICAM2, VWF, and RAB37 have been reported to be associated with T2DM or ASCVD-related diseases (48–60). Our study is the first to demonstrate that CD3D, TRAT1, and TMEEM244 are also associated with CAP progression in T2DM. Additionally, for non-coding RNA, we constructed a ceRNA regulatory network to further explore the progression mechanism of T2DM complicated with CAP.

The C-C motif chemokine receptor 7 (CCR7) was one of the major differentially expressed genes obtained by comparing T2DM patients with unstable CAP samples and non-T2DM patients with stable CAP. CCR7 is expressed by B cells, mature dendritic cells (DC), and several T cell subsets including immature, regulatory, and central memory T cells (61). Notably, CCR7 has been identified as a marker for AS progression (51). Similarly, CCR7 has been identified as a differentially expressed gene in non-DM and DM islet samples, indicating that CCR7 may play an important role in the pathogenesis of T2DM (52). Chemokines are a large family of proteins that regulate immune cell transport. They play a key role in guiding the movement and activity of leukocytes during homeostasis, immune surveillance, and inflammation. CC-chemokine ligand 19 (CCL19) and CC-chemokine ligand 21 (CCL21), are CCR7 ligands (61). Adaptive immunity is involved in the pathogenesis of AS, and the chemokines CCL19 and CCL21 are involved in lymphocyte homing in atherosclerotic lesions (48). A few studies have noticed elevated plasma levels of CCL19 and CCL21 in ApoE-/- mice with AS lesions, human CAP, and patients with coronary artery disease (49). Further, while the levels of CCL19 and CCL21 in the plasma in atherosclerotic lesions have been noticed, both these molecules are reported to be upregulated in carotid AS (50). Overall, a large number of studies have probed the role of chemokines and chemokine receptors in the development of AS (48) and by now the importance of chemokines in AS treatment is widely accepted (53). In view of this evidence, we believe that CCR7 is an important biomarker for the progression of T2DM complicated by CAP and plays an important role in identifying relevant targets for the treatment of the disease.

Intercellular adhesion molecule-2 (ICAM-2) was another differentially expressed gene obtained by comparison between T2DM patients with unstable CAP samples and non-T2DM patients with stable CAP. The protein encoded by this gene is a member of the intercellular adhesion molecule (ICAM) family. Numerous studies have shown that cell adhesion molecules (CAM) play a crucial role in AS initiation and progression (54, 55). ICAM-1 is widely recognized as the driver of the inflammatory response (62). One study has shown that ICAM-1 and ICAM-2 are involved in every step of neutrophil extravasation (56). A recent meta-analysis showed that elevated circulating CAM levels increased the risk of T2DM in a dose-dependent manner (57). Therefore, we suggest that ICAM-2 likely plays an important role in the progression of CAP-complicated T2DM.

Along with ICAM-2, the von Willebrand factor (VWF) was listed as another differentially expressed gene. VWF encodes a glycoprotein that is involved in hemostasis. VWF is engaged in primary hemostasis and coagulation processes, where it acts as a carrier for blood clotting factor VIII, prevents degradation of the factor by protein C, and significantly increases its plasma half-life (58). VWF plays a crucial role in platelet adhesion at sites of vascular injury. It mediates the initial progression of thrombosis at endothelial injury sites through specific interactions with the subendothelial collagen and platelet receptors (63, 64). Earlier studies have reported that VWF can be used as a procoagulant biomarker to predict the risk of cardiovascular and renal complications in diabetic patients (58). A meta-analysis characterized the prognostic value of VWF for ASCVD complications in T2DM patients by comparing plasma VWF levels in T2DM patients with and without coronary artery disease (59). In our study, VWF expression was significantly upregulated in T2DM patients with unstable CAP. Therefore, VWF has potential to be considered as an important biomarker of CAP progression in T2DM.

RAB37, a member of the RAS oncogene family, encodes a protein. Bioinformatics analysis has shown that RAB37 is differentially expressed in Alzheimer’s disease (65). Further, a proteomic study reported that the RAB37 protein is related to insulin secretory granules, which are responsible for the storage and secretion of insulin (60). In addition, RAB37 is expressed in human islets and β-cell lines and is involved in the regulation of insulin secretion (66). These studies indicate that RAB37 is associated with T2DM, and our study concurs with this evidence and indicates that RAB37 may play a role in T2DM with CAP at the same time.

The protein encoded by the CD3D gene is a part of the T-cell receptor (TCR)/CD3 complex and participates in T-cell development and signal transduction (67). It exists on the surface of T lymphocytes and plays an important role in the adaptive immune response.A bioinformatis study on the progression of rheumatoid arthritis has shown that CD3D is a potential key mediator and diagnostic marker (68). TRAT 1 stabilizes the TCR/CD3 complex on the surface of T cells. CD3D and TRAT1 are closely related to T cells, and studies have shown that T cells are involved in the pathogenesis of AS and T2DM (37, 69). TMEM244 is a protein-coding gene. Studies have shown that this gene may be used as a marker of Sézary syndrome and other T-cell lymphomas (70). Therefore, it is necessary to consider that the three genes identified for the first time in our study may participate in the progression of T2DM complicated with CAP through an immune response.

The ceRNA hypothesis has revealed a new mechanism for RNA interactions that suggests biological processes can be regulated by an intrinsic mechanism (71). Many bioinformatics studies have constructed AS- or T2DM-related ceRNA regulatory networks using the ceRNA hypothesis (72–74). In this study, a circRNA/lncRNA-miRNA-mRNA regulatory network with CAP progression in T2DM was constructed for the first time, including five circRNAs,13 lncRNAs, 46 miRNAs, and 54 mRNA. Our results suggest that multiple regulatory axes in ceRNA networks may play key roles in the pathogenesis of the disease. We propose that C22orf34—hsa-miR-6785-5p—RAB37, hsacirc_ 013887—hsa-miR-6785-5p/hsa-miR-4763-5p/hsa-miR-30b-3p-RAB37, MIR4435-1HG—hsa-miR-30b-3p—RAB37, and GAS5—hsa-miR-30b-3p—RAB37 are potential RNA regulatory pathways that regulate disease progression.

Previous studies have assessed the role of RNAs that are associated with T2DM and CAP progression (75, 76). However, only a few studies (13), including this study, have explored the progression of T2DM with CAP using advanced bioinformatic methods. While our results provide useful insights into the role of certain genes and RNAs in the progression of T2DM with CAP, our study also has a few limitations. First, there are few public databases that met the purpose of this study. Consequently, the sample size of RNA-seq in our study was small, and further research should be carried out using larger samples. Second, the diagnostic efficacy of some central genes and non-coding RNAs described in our study needs to be verified by clinical data. Finally, the ceRNA regulatory network based on bioinformatics prediction must be verified using molecular methods. In conclusion, the results of our study show that some potentially important genes, non-coding RNAs, pathways, and ceRNA regulatory networks are associated with the progression of T2DM with CAP. Based on these results, we predict that these observations will aid future studies involved in diagnosis, pathogenesis, and potential targeted therapy of T2DM with CAP.

## Data availability statement

Publicly available datasets were analyzed in this study. This data can be found here: https://www.ncbi.nlm.nih.gov/sra/PRJNA752896; https://www.ncbi.nlm.nih.gov/geo/query/acc.cgi?acc=GSE118481.

## Ethics statement

The studies involving human participants were reviewed and approved by the Ethics Committee of the First Hospital of Jilin University. The patients/participants provided their written informed consent to participate in this study.

## Author contributions

TY, MB, and BX were involved in data collection, data analysis, and article writing. RL designed the project and was responsible for article revisions. YG and QZ were involved in constructing the figures. XZ and TY were responsible for completing RT-qPCR. All authors contributed to the article and approved the submitted version.

## Funding

This work was supported by grants from the National Natural Science Foundation of China (81900739), Department of Science and Technology of Jilin Province (20210204217YY), Interdisciplinary Innovation Project of the First Hospital of Jilin University (JDYYJCHX2020014), and Scientific Research Project of the Jilin Provincial Department of Education (JJKH20211162KJ,3D5212598430).

## Acknowledgments

We would like to thank all patients and participants who contributed to this study. In addition, the scientific research center of China-Japan Union Hospital of Jilin University provided support for the RT-qPCR experiment.

## Conflict of interest

The authors declare that the research was conducted in the absence of any commercial or financial relationships that could be construed as potential conflict of interest.

## Publisher’s note

All claims expressed in this article are solely those of the authors and do not necessarily represent those of their affiliated organizations, or those of the publisher, the editors and the reviewers. Any product that may be evaluated in this article, or claim that may be made by its manufacturer, is not guaranteed or endorsed by the publisher.

## References

[1] Zheng Y Ley SH Hu FB. Global aetiology and epidemiology of type 2 diabetes mellitus and its complications. Nat Rev Endocrinol (2018) 14(2):88–98. doi: 10.1038/nrendo.2017.151 29219149

[2] Libby P Buring JE Badimon L Hansson GK Deanfield J Bittencourt MS. Atherosclerosis. Nat Rev Dis Primers (2019) 5(1):56. doi: 10.1038/s41572-019-0106-z 31420554

[3] Geovanini GR Libby P. Atherosclerosis and inflammation: Overview and updates. Clin Sci (Lond) (2018) 132(12):1243–52. doi: 10.1042/CS20180306 29930142

[4] Ooi YC Gonzalez NR. Management of extracranial carotid artery disease. Cardiol Clin (2015) 33(1):1–35. doi: 10.1016/j.ccl.2014.09.001 25439328 PMC4694631

[5] Stary HC. Natural history and histological classification of atherosclerotic lesions: An update. Arterioscler Thromb Vasc Biol (2000) 20(5):1177–8. doi: 10.1161/01.atv.20.5.1177 10807728

[6] Spagnoli LG Mauriello A Sangiorgi G Fratoni S Bonanno E Schwartz RS. Extracranial thrombotically active carotid plaque as a risk factor for ischemic stroke. JAMA (2004) 292(15):1845–52. doi: 10.1001/jama.292.15.1845 15494582

[7] Sarwar N Gao P Seshasai SR Gobin R Kaptoge S Di Angelantonio E. Diabetes mellitus, fasting blood glucose concentration, and risk of vascular disease: A collaborative meta-analysis of 102 prospective studies. Lancet (2010) 375(9733):2215–22. doi: 10.1016/s0140-6736(10)60484-9 PMC2904878 20609967

[8] Moreno PR Fuster V. New aspects in the pathogenesis of diabetic atherothrombosis. J Am Coll Cardiol (2004) 44(12):2293–300. doi: 10.1016/j.jacc.2004.07.060 15607389

[9] Burke AP Kolodgie FD Zieske A Fowler DR Weber DK Varghese PJ. Morphologic findings of coronary atherosclerotic plaques in diabetics: A postmortem study. Arterioscler Thromb Vasc Biol (2004) 24(7):1266–71. doi: 10.1161/01.ATV.0000131783.74034.97 15142859

[10] Kato K Yonetsu T Kim SJ Xing L Lee H McNulty I. Comparison of nonculprit coronary plaque characteristics between patients with and without diabetes: A 3-vessel optical coherence tomography study. JACC Cardiovasc Interv (2012) 5(11):1150–8. doi: 10.1016/j.jcin.2012.06.019 23174639

[11] Sun B Li X Liu X Ge X Lu Q Zhao X. Association between carotid plaque characteristics and acute cerebral infarction determined by mri in patients with type 2 diabetes mellitus. Cardiovasc Diabetol (2017) 16(1):111. doi: 10.1186/s12933-017-0592-9 28893252 PMC5594451

[12] Li X Sun B Wang L Zhang J Zhang J Zhao Z. Association of type 2 diabetes mellitus and glycemic control with intracranial plaque characteristics in patients with acute ischemic stroke. J Magn Reson Imaging (2021) 54(2):655–66. doi: 10.1002/jmri.27614 33786939

[13] Li YY Zhang S Wang H Zhang SX Xu T Chen SW. Identification of crucial genes and pathways associated with atherosclerotic plaque in diabetic patients. Pharmgenomics Pers Med (2021) 14:211–20. doi: 10.2147/PGPM.S281705 PMC7869704 33568933

[14] Bao MH Zhang RQ Huang XS Zhou J Guo Z Xu BF. Transcriptomic and proteomic profiling of human stable and unstable carotid atherosclerotic plaques. Front Genet (2021) 12:755507. doi: 10.3389/fgene.2021.755507 34804124 PMC8599967

[15] Barrett T Wilhite SE Ledoux P Evangelista C Kim IF Tomashevsky M. Ncbi geo: Archive for functional genomics data sets–update. Nucleic Acids Res (2013) 41(Database issue):D991–5. doi: 10.1093/nar/gks1193 PMC3531084 23193258

[16] Chen G Li Y Su Y Zhou L Zhang H Shen Q. Identification of candidate genes for necrotizing enterocolitis based on microarray data. Gene (2018) 661:152–9. doi: 10.1016/j.gene.2018.03.088 29605607

[17] Ritchie ME Phipson B Wu D Hu Y Law CW Shi W. Limma powers differential expression analyses for rna-sequencing and microarray studies. Nucleic Acids Res (2015) 43(7):e47. doi: 10.1093/nar/gkv007 25605792 PMC4402510

[18] Wang L Cao C Ma Q Zeng Q Wang H Cheng Z. Rna-seq analyses of multiple meristems of soybean: Novel and alternative transcripts, evolutionary and functional implications. BMC Plant Biol (2014) 14:169. doi: 10.1186/1471-2229-14-169 24939556 PMC4070088

[19] Kanehisa M Goto S. Kegg: Kyoto encyclopedia of genes and genomes. Nucleic Acids Res (2000) 28(1):27–30. doi: 10.1093/nar/28.1.27 10592173 PMC102409

[20] Huang DW Sherman BT Lempicki RA. Systematic and integrative analysis of Large gene lists using David bioinformatics resources. Nat Protoc (2009) 4(1):44–57. doi: 10.1038/nprot.2008.211 19131956

[21] Huang da W Sherman BT Lempicki RA. Bioinformatics enrichment tools: Paths toward the comprehensive functional analysis of Large gene lists. Nucleic Acids Res (2009) 37(1):1–13. doi: 10.1093/nar/gkn923 19033363 PMC2615629

[22] Consortium GO. The gene ontology (Go) project in 2006. Nucleic Acids Res (2006) 34(Database issue):D322–6. doi: 10.1093/nar/gkj021 PMC1347384 16381878

[23] Szklarczyk D Gable AL Nastou KC Lyon D Kirsch R Pyysalo S. The string database in 2021: Customizable protein-protein networks, and functional characterization of user-uploaded Gene/Measurement sets. Nucleic Acids Res (2021) 49(D1):D605–D12. doi: 10.1093/nar/gkaa1074 PMC7779004 33237311

[24] Shannon P Markiel A Ozier O Baliga NS Wang JT Ramage D. Cytoscape: A software environment for integrated models of biomolecular interaction networks. Genome Res (2003) 13(11):2498–504. doi: 10.1101/gr.1239303 PMC403769 14597658

[25] Scardoni G Petterlini M Laudanna C. Analyzing biological network parameters with centiscape. Bioinformatics (2009) 25(21):2857–9. doi: 10.1093/bioinformatics/btp517 PMC2781755 19729372

[26] Maere S Heymans K Kuiper M. Bingo: A cytoscape plugin to assess overrepresentation of gene ontology categories in biological networks. Bioinformatics (2005) 21(16):3448–9. doi: 10.1093/bioinformatics/bti551 15972284

[27] Han H Cho JW Lee S Yun A Kim H Bae D. Trrust V2: An expanded reference database of human and mouse transcriptional regulatory interactions. Nucleic Acids Res (2018) 46(D1):D380–D6. doi: 10.1093/nar/gkx1013 PMC5753191 29087512

[28] Zou KH Tuncali K Silverman SG. Correlation and simple linear regression. Radiology (2003) 227(3):617–22. doi: 10.1148/radiol.2273011499 12773666

[29] Betel D Koppal A Agius P Sander C Leslie C. Comprehensive modeling of microrna targets predicts functional non-conserved and non-canonical sites. Genome Biol (2010) 11(8):R90. doi: 10.1186/gb-2010-11-8-r90 20799968 PMC2945792

[30] Hemmat N Mokhtarzadeh A Aghazadeh M Jadidi-Niaragh F Baradaran B Baghi HB. Role of micrornas in epidermal growth factor receptor signaling pathway in cervical cancer. Mol Biol Rep (2020) 47(6):4553–68. doi: 10.1007/s11033-020-05494-4 32383136

[31] Chen HJ Tas SW de Winther MPJ. Type-I interferons in atherosclerosis. J Exp Med (2020) 217(1):e20190459. doi: 10.1084/jem.20190459 31821440 PMC7037237

[32] Wesseling M Sakkers TR de Jager SCA Pasterkamp G Goumans MJ. The morphological and molecular mechanisms of Epithelial/Endothelial-to-Mesenchymal transition and its involvement in atherosclerosis. Vascul Pharmacol (2018) 106:1–8. doi: 10.1016/j.vph.2018.02.006 29471141

[33] Nigro J Osman N Dart AM Little PJ. Insulin resistance and atherosclerosis. Endocr Rev (2006) 27(3):242–59. doi: 10.1210/er.2005-0007 16492903

[34] Einarson TR Acs A Ludwig C Panton UH. Prevalence of cardiovascular disease in type 2 diabetes: A systematic literature review of scientific evidence from across the world in 2007-2017. Cardiovasc Diabetol (2018) 17(1):83. doi: 10.1186/s12933-018-0728-6 29884191 PMC5994068

[35] Ray A Huisman MV Tamsma JT van Asten J Bingen BO Broeders EA. The role of inflammation on atherosclerosis, intermediate and clinical cardiovascular endpoints in type 2 diabetes mellitus. Eur J Intern Med (2009) 20(3):253–60. doi: 10.1016/j.ejim.2008.07.008 19393492

[36] Ma SD Mussbacher M Galkina EV. Functional role of b cells in atherosclerosis. Cells (2021) 10(2):270. doi: 10.3390/cells10020270 33572939 PMC7911276

[37] Saigusa R Winkels H. Ley K. T Cell Subsets Functions Atherosclerosis. Nat Rev Cardiol (2020) 17(7):387–401. doi: 10.1038/s41569-020-0352-5 32203286 PMC7872210

[38] Zhou T Hu Z Yang S Sun L Yu Z Wang G. Role of adaptive and innate immunity in type 2 diabetes mellitus. J Diabetes Res (2018) 2018:7457269. doi: 10.1155/2018/7457269 30533447 PMC6250017

[39] SantaCruz-Calvo S Bharath L Pugh G SantaCruz-Calvo L Lenin RR Lutshumba J. Adaptive immune cells shape obesity-associated type 2 diabetes mellitus and less prominent comorbidities. Nat Rev Endocrinol (2022) 18(1):23–42. doi: 10.1038/s41574-021-00575-1 34703027 PMC11005058

[40] Edgar L Akbar N Braithwaite AT Krausgruber T Gallart-Ayala H Bailey J. Hyperglycemia induces trained immunity in macrophages and their precursors and promotes atherosclerosis. Circulation (2021) 144(12):961–82. doi: 10.1161/CIRCULATIONAHA.120.046464 PMC8448412 34255973

[41] Wu MY Li CJ Hou MF Chu PY. New insights into the role of inflammation in the pathogenesis of atherosclerosis. Int J Mol Sci (2017) 18(10):2034. doi: 10.3390/ijms18102034 PMC5666716 28937652

[42] Herrero-Fernandez B Gomez-Bris R Somovilla-Crespo B Gonzalez-Granado JM. Immunobiology of atherosclerosis: A complex net of interactions. Int J Mol Sci (2019) 20(21):5293. doi: 10.3390/ijms20215293 PMC6862594 31653058

[43] Ketelhuth DFJ Lutgens E Bäck M Binder CJ Van den Bossche J Daniel C. Immunometabolism and atherosclerosis: Perspectives and clinical significance: A position paper from the working group on atherosclerosis and vascular biology of the European society of cardiology. Cardiovasc Res (2019) 115(9):1385–92. doi: 10.1093/cvr/cvz166 PMC6681176 31228191

[44] Tan L Xu Q Shi R Zhang G. Bioinformatics analysis reveals the landscape of immune cell infiltration and immune-related pathways participating in the progression of carotid atherosclerotic plaques. Artif Cells Nanomed Biotechnol (2021) 49(1):96–107. doi: 10.1080/21691401.2021.1873798 33480285

[45] Li S Zhang Q Huang Z Tao W Zeng C Yan L. Comprehensive analysis of immunocyte infiltration and the key genes associated with intraplaque hemorrhage in carotid atherosclerotic plaques. Int Immunopharmacol (2022) 106:108633. doi: 10.1016/j.intimp.2022.108633 35183915

[46] Koelwyn GJ Corr EM Erbay E Moore KJ. Regulation of macrophage immunometabolism in atherosclerosis. Nat Immunol (2018) 19(6):526–37. doi: 10.1038/s41590-018-0113-3 PMC6314674 29777212

[47] Shen Y Xu LR Tang X Lin CP Yan D Xue S. Identification of potential therapeutic targets for atherosclerosis by analysing the gene signature related to different immune cells and immune regulators in atheromatous plaques. BMC Med Genomics (2021) 14(1):145. doi: 10.1186/s12920-021-00991-2 34082770 PMC8176741

[48] Li J Ley K. Lymphocyte migration into atherosclerotic plaque. Arterioscler Thromb Vasc Biol (2015) 35(1):40–9. doi: 10.1161/atvbaha.114.303227 PMC4429868 25301842

[49] Damås JK Smith C Øie E Fevang B Halvorsen B Waehre T. Enhanced expression of the homeostatic chemokines Ccl19 and Ccl21 in clinical and experimental atherosclerosis: Possible pathogenic role in plaque destabilization. Arterioscler Thromb Vasc Biol (2007) 27(3):614–20. doi: 10.1161/01.ATV.0000255581.38523.7c 17170367

[50] Halvorsen B Dahl TB Smedbakken LM Singh A Michelsen AE Skjelland M. Increased levels of Ccr7 ligands in carotid atherosclerosis: Different effects in macrophages and smooth muscle cells. Cardiovasc Res (2014) 102(1):148–56. doi: 10.1093/cvr/cvu036 24518141

[51] Hueso M Mallén A Casas Á Guiteras J Sbraga F Blasco-Lucas A. Integrated Mirna/Mrna counter-expression analysis highlights oxidative stress-related genes Ccr7 and Foxo1 as blood markers of coronary arterial disease. Int J Mol Sci (2020) 21(6):1943. doi: 10.3390/ijms21061943 PMC7139611 32178422

[52] Cui Y Chen W Chi J Wang L. Differential expression network analysis for diabetes mellitus type 2 based on expressed level of islet cells. Ann Endocrinol (Paris) (2016) 77(1):22–9. doi: 10.1016/j.ando.2015.11.002 26874994

[53] Koenen RR Weber C. Chemokines: Established and novel targets in atherosclerosis. EMBO Mol Med (2011) 3(12):713–25. doi: 10.1002/emmm.201100183 PMC3377113 22038924

[54] Ling S Nheu L Komesaroff PA. Cell adhesion molecules as pharmaceutical target in atherosclerosis. Mini Rev Med Chem (2012) 12(2):175–83. doi: 10.2174/138955712798995057 22070689

[55] Chi Z Melendez AJ. Role of cell adhesion molecules and immune-cell migration in the initiation, onset and development of atherosclerosis. Cell Adh Migr (2007) 1(4):171–5. doi: 10.4161/cam.1.4.5321 PMC2634102 19262139

[56] Lyck R Enzmann G. The physiological roles of icam-1 and icam-2 in neutrophil migration into tissues. Curr Opin Hematol (2015) 22(1):53–9. doi: 10.1097/moh.0000000000000103 25427141

[57] Qiu S Cai X Liu J Yang B Zügel M Steinacker JM. Association between circulating cell adhesion molecules and risk of type 2 diabetes: A meta-analysis. Atherosclerosis (2019) 287:147–54. doi: 10.1016/j.atherosclerosis.2019.06.908 31280040

[58] Domingueti CP Dusse LM Carvalho M de Sousa LP Gomes KB Fernandes AP. Diabetes mellitus: The linkage between oxidative stress, inflammation, hypercoagulability and vascular complications. J Diabetes Complications (2016) 30(4):738–45. doi: 10.1016/j.jdiacomp.2015.12.018 26781070

[59] Peng X Wang X Fan M Zhao J Lin L Liu J. Plasma levels of Von willebrand factor in type 2 diabetes patients with and without cardiovascular diseases: A meta-analysis. Diabetes Metab Res Rev (2020) 36(1):e3193. doi: 10.1002/dmrr.3193 31145835

[60] Brunner Y Couté Y Iezzi M Foti M Fukuda M Hochstrasser DF. Proteomics analysis of insulin secretory granules. Mol Cell Proteomics (2007) 6(6):1007–17. doi: 10.1074/mcp.M600443-MCP200 17317658

[61] Forster R Davalos-Misslitz AC Rot A. Ccr7 and its ligands: Balancing immunity and tolerance. Nat Rev Immunol (2008) 8(5):362–71. doi: 10.1038/nri2297 18379575

[62] Bui TM Wiesolek HL Sumagin R. Icam-1: A master regulator of cellular responses in inflammation, injury resolution, and tumorigenesis. J Leukoc Biol (2020) 108(3):787–99. doi: 10.1002/jlb.2mr0220-549r PMC7977775 32182390

[63] Domingueti CP Dusse LM Carvalho M Gomes KB Fernandes AP. Hypercoagulability and cardiovascular disease in diabetic nephropathy. Clin Chim Acta (2013) 415:279–85. doi: 10.1016/j.cca.2012.10.061 23159842

[64] Reininger AJ. Function of Von willebrand factor in haemostasis and thrombosis. Haemophilia (2008) 14 Suppl 5:11–26. doi: 10.1111/j.1365-2516.2008.01848.x 18786007

[65] Xu H Jia J. Immune-related hub genes and the competitive endogenous rna network in alzheimer's disease. J Alzheimers Dis (2020) 77(3):1255–65. doi: 10.3233/jad-200081 32925027

[66] Ljubicic S Bezzi P Brajkovic S Nesca V Guay C Ohbayashi N. The gtpase Rab37 participates in the control of insulin exocytosis. PLoS One (2013) 8(6):e68255. doi: 10.1371/journal.pone.0068255 23826383 PMC3694898

[67] Wang Q Li P Wu W. A systematic analysis of immune genes and overall survival in cancer patients. BMC Cancer (2019) 19(1):1225. doi: 10.1186/s12885-019-6414-6 31842801 PMC6915928

[68] Lu J Bi Y Zhu Y Huipeng S Duan W Zhou J. Cd3d, gzmk, and Klrb1 are potential markers for early diagnosis of rheumatoid arthritis, especially in anti-citrullinated protein antibody-negative patients. Front Pharmacol (2021) 12:726529. doi: 10.3389/fphar.2021.726529 34603038 PMC8483717

[69] Stentz FB Kitabchi AE. Activated T lymphocytes in type 2 diabetes: Implications from in vitro studies. Curr Drug Targets (2003) 4(6):493–503. doi: 10.2174/1389450033490966 12866664

[70] Iżykowska K Rassek K Żurawek M Nowicka K Paczkowska J Ziółkowska-Suchanek I. Hypomethylation of the promoter region drives ectopic expression of Tmem244 in sézary cells. J Cell Mol Med (2020) 24(18):10970–7. doi: 10.1111/jcmm.15729 PMC7521334 32794659

[71] Salmena L Poliseno L Tay Y Kats L Pandolfi PP. A cerna hypothesis: The Rosetta stone of a hidden rna language? Cell (2011) 146(3):353–8. doi: 10.1016/j.cell.2011.07.014 PMC3235919 21802130

[72] Zhao W Meng X Liang J. Analysis of circrna-mrna expression profiles and functional enrichment in diabetes mellitus based on high throughput sequencing. Int Wound J (2022) 19(5):1253–62. doi: 10.1111/iwj.13838 PMC9284653 35504843

[73] Zhang F Zhang R Zhang X Wu Y Li X Zhang S. Comprehensive analysis of circrna expression pattern and circrna-Mirna-Mrna network in the pathogenesis of atherosclerosis in rabbits. Aging (Albany NY) (2018) 10(9):2266–83. doi: 10.18632/aging.101541 PMC6188486 30187887

[74] Yu XH Deng WY Chen JJ Xu XD Liu XX Chen L. Lncrna Kcnq1ot1 promotes lipid accumulation and accelerates atherosclerosis *Via* functioning as a cerna through the mir-452-3p/Hdac3/Abca1 axis. Cell Death Dis (2020) 11(12):1043. doi: 10.1038/s41419-020-03263-6 33293505 PMC7723992

[75] Zhang R Ji Z Yao Y Zuo W Yang M Qu Y. Identification of hub genes in unstable atherosclerotic plaque by conjoint analysis of bioinformatics. Life Sci (2020) 262:118517. doi: 10.1016/j.lfs.2020.118517 33011223

[76] Massaro JD Polli CD Costa ESM Alves CC Passos GA Sakamoto-Hojo ET. Post-transcriptional markers associated with clinical complications in type 1 and type 2 diabetes mellitus. Mol Cell Endocrinol (2019) 490:1–14. doi: 10.1016/j.mce.2019.03.008 30926524

