# Supplementary figures and images for: Identification of potential biomarkers and pathways associated with carotid atherosclerotic plaques in type 2 diabetes mellitus: A transcriptomics study
Source: Front Endocrinol (Lausanne). 2022 Sep 16;13:981100. doi: 10.3389/fendo.2022.981100 (PMC9523108; doi:10.3389/fendo.2022.981100)

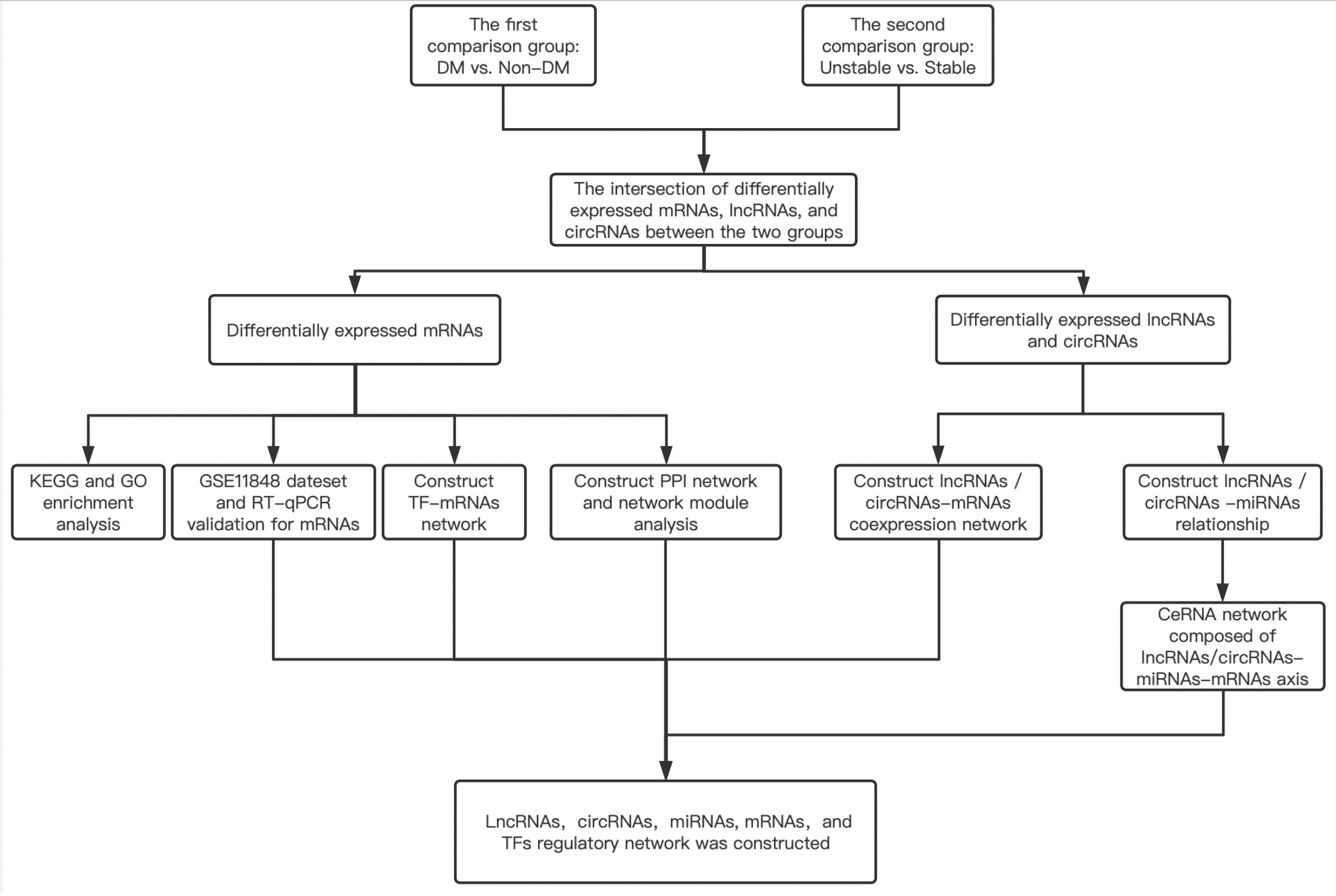

Supplement: Supplementary file 1 [file Image_1.jpeg]
