# Supplementary material for: Identification of potential biomarkers and pathways associated with carotid atherosclerotic plaques in type 2 diabetes mellitus: A transcriptomics study
Source: Front Endocrinol (Lausanne). 2022 Sep 16;13:981100. doi: 10.3389/fendo.2022.981100 (PMC9523108; doi:10.3389/fendo.2022.981100)
Supplement: Supplementary file 2 [file DataSheet_1.docx]

Supplementary Material

# Supplementary Figures and Tables

## Supplementary Figures

**Supplementary Figure 1.** Flow chart

## 1.2 Supplementary Tables

**Supplementary Table 1**. The primer sequences included in this study

**Supplementary Table 2.** Statistical table of the number of RNAs significantly differentially expressed in each comparison group

**Supplementary Table 3.** Go analysis (including BP, MF, CC) and KEGG signal pathway list related to 171 mRNAs

**Supplementary Table 4.** PPI network diagram top20 gene network node topology information table

**Supplementary Table 5.** All the significantly related biological process annotations in the four modules obtained by the PPI network

**Supplementary Table 6.** Connection relationship of comprehensive network diagram where important overlapping RNAs are located
