# Supplementary material for: Identification of potential biomarkers and pathways associated with carotid atherosclerotic plaques in type 2 diabetes mellitus: A transcriptomics study
Source: Front Endocrinol (Lausanne). 2022 Sep 16;13:981100. doi: 10.3389/fendo.2022.981100 (PMC9523108; doi:10.3389/fendo.2022.981100)
Supplement: Supplementary file 3 [file Table_1.docx]

**Supplementary** **Table 1** The primer sequences included in this study.

| Name | primer sequences (5’–3’) |
| --- | --- |
| RAB37 forward | GTGGATGGCGTGAGAGTGAAGC |
| RAB37 reverse | TGATGTCATACAGCAGAAGCAAGGC |
| CD3D forward | TACTGGCTACCCTTCTCTCGCAAG |
| CD3D reverse | GTTCCCACCGTTCCCTCTACCC |
| TRAT1 forward | GCACAGGCAACCAATGAAACACAG |
| TRAT1 reverse | GTTTCCTGGGCTTTCTACGCTTCC |
| VWF forward | TGCGACACCATTGCTGCCTATG |
| VWF reverse | GCCACTCACACTCATACCCGTTC |
| TMEM244 forward | TCTCCAGGTCAGAGTTGCTCCAAG |
| TMEM244 reverse | CCATGCTCAGGGACACATAGTACAC |
| CCR7 forward | GCTGTGGTCGTGGTCTTCATAGTC |
| CCR7 reverse | AGGCGATGTTGAGTTGCTTACTGAG |
| ICAM2 forward | CGGATGAGAAGGTATTCGAGGT |
| ICAM2 reverse | CACCCACTTCAGGCTGGTTAC |
| β-Actin forward | CATGTACGTTGCTATCCAGGC |
| β-Actin reverse | CTCCTTAATGTCACGCACGAT |
